# The adiponectin-PPARγ axis in hepatic stellate cells regulates liver fibrosis

**DOI:** 10.1016/j.celrep.2024.115165

**Published:** 2025-01-09

**Authors:** Shangang Zhao, Qingzhang Zhu, Wang-Hsin Lee, Jan-Bernd Funcke, Zhuzhen Zhang, May-Yun Wang, Qian Lin, Bianca Field, Xue-Nan Sun, Guannan Li, Mbolle Ekane, Toshiharu Onodera, Na Li, Yi Zhu, Christine M. Kusminski, Terry D. Hinds, Philipp E. Scherer

**Affiliations:** 1 Touchstone Diabetes Center, University of Texas Southwestern Medical Center, Dallas, TX, USA; 2 Sam and Ann Barshop Institute for Longevity and Aging Studies, Division of Endocrinology, Department of Medicine and Department of Cellular & Integrative Physiology, University of Texas Health Science Center at San Antonio, San Antonio, TX 78229, USA; 3 Department of Pharmacology and Nutritional Sciences, University of Kentucky College of Medicine, Lexington, KY, USA; 4 College of Life Sciences, Taikang Center for Life and Medical Sciences, Wuhan University, Wuhan 430072, China; 5 Department of Endocrinology and Metabolism, Tianjin Medical University General Hospital, Tianjin 300052, China; 6 Children’s Nutrition Research Center, Department of Pediatrics, Baylor College of Medicine, Houston, TX 77030, USA; 7 Barnstable Brown Diabetes and Obesity Center, University of Kentucky, Lexington, KY 40508, USA; 8 Department of Adipose Management, Osaka University Graduate School of Medicine, Osaka, Japan

## Abstract

Hepatic stellate cells (HSCs) are key drivers of local fibrosis. Adiponectin, conventionally thought of as an adipokine, is also expressed in quiescent HSCs. However, the impact of its local expression on the progression of liver fibrosis remains unclear. We recently generated a transgenic mouse line (Lrat-rtTA) that expresses the doxycycline-responsive transcriptional activator rtTA under the control of the HSC-specific *lecithin retinol acyltransferase* (*Lrat*) promoter, which enables us to specifically and inducibly overexpress or eliminate genes in these cells. The inducible elimination of HSCs protects mice from methionine/choline-deficient (MCD) diet-induced liver fibrosis, confirming their causal involvement in fibrosis development. We generated HSC-specific adiponectin overexpression and null models that demonstrate that HSC-specific adiponectin overexpression dramatically reduces liver fibrosis, whereas HSC-specific adiponectin elimination accelerates fibrosis progression. We identify a local adiponectin-peroxisome proliferator-activated receptor gamma (PPARγ) axis in HSCs that exerts a marked influence on the progression of local fibrosis, independent of circulating adiponectin derived from adipocytes.

## In brief

Zhao et al. modulate adiponectin levels produced in hepatic stellate cells. Local expression of adiponectin in HSCs prevents the development of liver fibrosis, while the deletion of adiponectin in HSCs accelerates its development. An increase of PPARγ activity in HSCs partially recaptures the phenotype of adiponectin overexpression in HSCs.

## Graphical Abstract

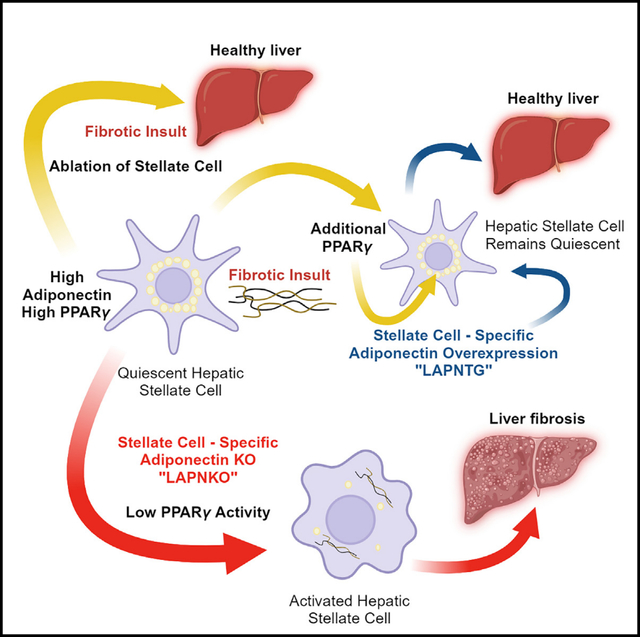

## INTRODUCTION

Liver fibrosis, induced by chronic viral or metabolic stress, is a severe metabolic disorder that can progress to liver cirrhosis and hepatocellular carcinoma (HCC).^1^ The pathological sequelae of this fibrotic state are a leading cause of mortality, globally with more than one million deaths annually. Currently, no effective antifibrotic therapies are available. Hepatic stellate cells (HSCs) are widely viewed as crucial contributors to liver fibrosis. HSCs form clusters of cells located in the perisinusoidal space of the liver. In response to liver injury, HSCs transit from a quiescent to an activated state and start producing large amounts of collagen.^1,2^ Consequently, strategies that prevent the activation of HSCs or revert them to the quiescent state may hold potential in the treatment of liver fibrosis.

As one of the most widely studied adipokines, adiponectin exerts pleotropic effects regulating energy homeostasis. Elevated adiponectin levels are associated with enhanced insulin sensitivity and reduced systemic inflammation,^3–5^ while a lack of adiponectin results in diverse metabolic aberrations. Of note, we and others have also implicated adiponectin as a regulator of liver fibrosis.^6,7^ HSCs express adiponectin receptors, and adiponectin displays robust anti-fibrotic actions in the liver that involve blocking HSC activation. Previous reports have indicated that a systemic lack of adiponectin enhances carbon tetrachloride (CCl_4_)-induced liver fibrosis,^6,8^ whereas elevating adiponectin signaling by administrating the adiponectin receptor agonist ADP355 is sufficient to attenuate it.^9^ Furthermore, we have recently shown that aging adiponectin-deficient mice develop severe liver fibrosis despite a lack of local lipid droplet accumulation, suggesting that the development of liver fibrosis does not necessarily involve steatosis.^10^ Some *in vitro* observations have indicated that adiponectin reduces HSC migration by promoting tissue inhibitor of metalloproteinase 1 secretion and inducing nitric oxide production.^11,12^ These observations highlight the pivotal role of adiponectin in regulating liver fibrosis.

Adipocytes are the major source of circulating adiponectin. However, several other cell types including HSCs, cardiomyocytes, and certain cells in the kidney may express adiponectin as well.^13–16^ Still, it remains uncertain if the production of adiponectin by HSCs impacts local fibrotic processes or whether the observed beneficial effects of adiponectin are solely due to its circulating pool. Using freshly isolated murine HSCs, we have previously shown that the continuous decline of adiponectin expression that occurs with the prolonged *in vitro* culture of these cells also coincides with a robust induction of fibrotic gene expression.^8^ This suggests a direct involvement of HSC-derived adiponectin production in HSC activation. However, due to the lack of transgenic tools to target HSCs *in vivo*, the relevance of adiponectin expression in HSCs for the progression of liver fibrosis has remained unexplored.

Here, to address this shortage of transgenic tools, we generated a transgenic mouse line (Lrat-rtTA) that harbors a *lecithin retinol acyltransferase* (*Lrat*) promoter-driven rtTA cassette. We used this mouse line to establish HSC-specific, inducible adiponectin overexpression and elimination models. Taking advantage of these two as well as other mouse models, we demonstrate a crucial role of local adiponectin production in HSCs in the regulation of liver fibrosis via peroxisome proliferator-activated receptor gamma (PPARγ).

## RESULTS

### The expression of adiponectin and fibrotic genes is inversely correlated in HSCs

**Figure 1.**
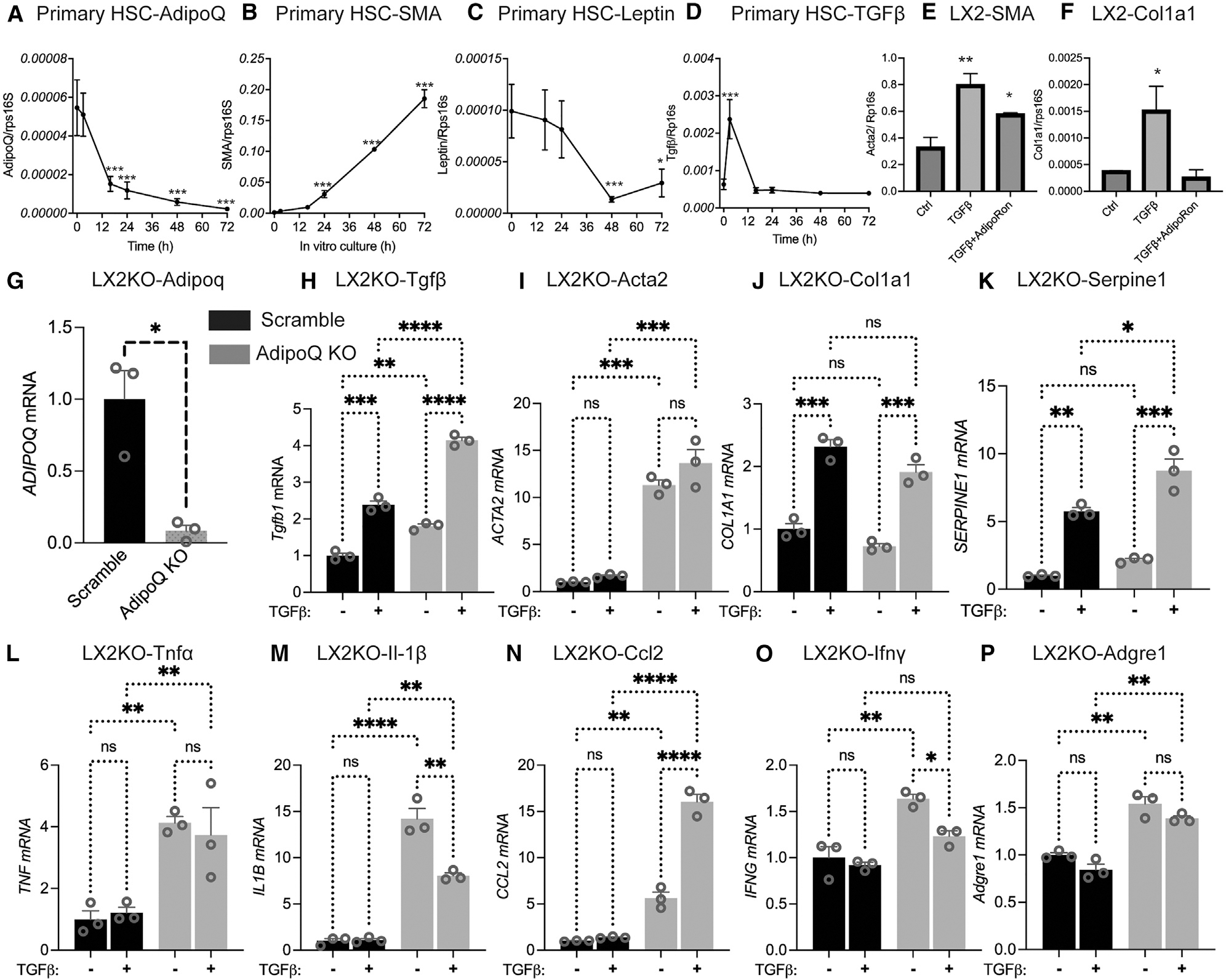
Adiponectin expression in HSCs is negatively associated with their activation (A–D) Freshly isolated mouse HSCs were cultured for different times. mRNA expression of (A) adiponectin, (B) smooth muscle actin, (C) leptin, and (D) TGF-β (*n* = 3). (E and F) LX2 cells were starved in serum-free medium overnight and then treated with 20 ng/mL TGF-β and/or 5 μM AdipoRon for 24 h. mRNA expression of (E) smooth muscle actin and (F) COL1A1 (*n* = 3). (G–P) Ctrl and adiponectin knockout LX2 cell lines were treated in the presence and absence of TGF-β. (G) Expression of adiponectin was determined. Fibrotic markers, including (H) TGF-β, (I) Acta 2, (J) Col1a1, and (K) Serpine, and inflammation markers, including (L) TNFα, (M) IL-1β, (N) Ccl2, (O) Ifnγ, and (P) Adgre1, were measured. Data are displayed as mean ± SEM and analyzed by unpaired Student’s t test for all the figures: *p < 0.05, **p < 0.01, ***p < 0.001.

Activated HSCs are pivotal sources of collagen and drivers of liver fibrosis. The factors that govern the switch of HSCs from a quiescent to an activated state are largely unknown. To assess whether local HSC-derived adiponectin is involved in this process, we isolated HSCs from wild-type mice and examined the gene expression of adiponectin as well as fibrotic genes after different times in culture. Strikingly, we observed a rapid and pronounced decline of adiponectin expression within as early as 2 h of culture (Figure 1A), while the expression of fibrotic genes such as smooth muscle actin (SMA) rapidly increased (Figure 1B). Of note, this decline of adiponectin expression occurred prior to the onset of SMA induction, suggesting that adiponectin may suppress the fibrotic gene expression in activated HSCs. In contrast to adiponectin, leptin expression (found at only low levels in stellate cells) decreased at a substantially slower rate over time (Figure 1C). Transforming growth factor β (TGF-β) expression displayed dynamic changes with a short-lived peak at 2 h of culture and subsequent return to basal levels (Figure 1D). The transient induction of TGF-β at the 2-h point may indeed be due to isolation- and culture-associated transition of the HSCs from the quiescent to the activated state.

To further examine the role of adiponectin in HSC activation, we took advantage of the human HSC line LX2. Treatment of LX2 cells with TGF-β significantly increased the expression of fibrotic maker genes such as SMA and COL1A1 (Figures 1E and 1F). Elevating adiponectin signaling by the addition of the adiponectin receptor agonist AdipoRon effectively suppressed these TGF-β-induced changes in gene expression (Figures 1E and 1F). To further examine the role of adiponectin in liver fibrosis, we knocked down adiponectin in LX2 cell line using CRISPR-Cas9 technology. We found that the expression of adiponectin in LX2 knockout (LX2 KO) cell line was reduced by more than 90%, compared to its expression in cells with a scrambled construct (Ctrl) (Figure 1G). Then we exposed Ctrl and LX2 KO cells with the presence or absence of TGF-β. While we found that TGF-β significantly increased the expression of fibrotic genes, including Tgfβ itself, Acta2, Col1a1, and Serpine1, both in Ctrl and LX2 KO cells, the increase at baseline and after TGF-β treatment is higher in the LX2 KO cells (Figures 1H–1K). Hence, deletion of adiponectin exacerbated the fibrotic gene expression (Figures 1H–1K). Particularly, even without TGF-β treatment, the deletion of adiponectin in LX2 cells was sufficient to induce significant increases in fibrotic gene expression for Tgfβ and Acta2 (Figures 1H and 1I). Taken together, these observations highlight the importance of adiponectin in the regulation of HSC activation and fibrotic gene expression.

As adiponectin has been shown to potently inhibit inflammation in other contexts, we assessed whether the deletion of adiponectin in LX2 cells affects inflammatory gene expression. Using the same cell lines, we examined several inflammatory markers. We found that the deletion of adiponectin in LX2 cells greatly induced inflammatory gene expression, including TNFα, IL-1β, Ccl2, Ifnγ, and Adgre1, even in the absence of TGF-β exposure (Figures 1L–1P). These *in vitro* results refect the powerful impact that adiponectin exerts on both inflammatory and fibrotic gene expression.

### Generation and characterization of the Lrat-rtTA mouse line

HSCs are a major site of retinoid storage and highly enriched in the expression of *lecithin retinol acyltransferase* (*Lrat*) (Figure S1A). We took advantage of this enriched expression to generate a mouse model in which the expression of the doxycycline-responsive transcriptional activator rtTA is driven by the *Lrat* promoter (Lrat-rtTA). To validate the Lrat-rtTA line, we first assessed the expression of rtTA in several different cell types and tissues, including HSCs, liver, subcutaneous adipose tissue, heart, kidney, cortex, hippocampus, cerebellum, duodenum, jejunum, ileum, colon, eye, and testis. Among all the tissues examined, we detected a low-level expression of rtTA in duodenum and jejunum. However, Lrat expression in HSCs was 1,000-fold higher compared to other tissues, confirming the specificity of the Lrat promoter (Figure S1A). The expression of rtTA was aligned with the expression of Lrat in different cells and tissues, with the highest expression in HSCs (Figure S1B). We moreover confirmed that adiponectin is locally expressed in HSCs, while being completely undetectable in the liver as a whole (Figure S1C). To further characterize the Lrat-rtTA line, we crossed it to a TRE-GFP reporter line (Figure S1D) and fed these mice doxycycline (DOX)-containing chow (chow+DOX). Immunohistochemistry of collected livers revealed an overlapping expression of GFP and adiponectin (Figure S1E), indicating that our Lrat-rtTA line specifically targets HSCs and their manipulation in diverse ways.

### Ablation of HSCs prevents MCD diet-induced liver fibrosis

As HSCs are the major players in liver fibrosis and contribute to more than 90% of collagen deposition during liver injury, we hypothesized that ablation of HSCs would protect mice from diet-induced liver fibrosis. To this end, we crossed the Lrat-rtTA line with the previously established TRE-ATTAC line (Tre-Cas8, Figure S2A). The ATTAC cassette contains a caspase-8 moiety that can be activated by the addition of a small-molecule dimerizer, resulting in an efficient induction of apoptotic cell death.^17–19^ In the presence of doxycycline and the dimerizer, the ATTAC approach eliminates most HSCs. Using the LATTAC model, we first performed HSC elimination and then placed the mice on a methionine/choline-deficient (MCD) diet to induce liver fibrosis.^20^ We observed no significant differences in either the body weight development or glucose intolerance of LATTAC and control mice (Figures S2B and S2C). These observations indicate that HSCs play only a minor role in regulating systemic metabolism under the given conditions. Trichrome staining of the liver, however, revealed a drastic reduction in liver fibrosis in LATTAC mice (Figure S2D). Of special interest, we noticed that the removal of HSCs in LATTAC mice also greatly reduced liver steatosis. These observations strongly support the importance of HSCs for the progression of fibrotic processes in the liver.

### Overexpression of adiponectin in HSCs protects mice from thermoneutrality- and chronic ER stress-induced liver fibrosis

**Figure 2.**
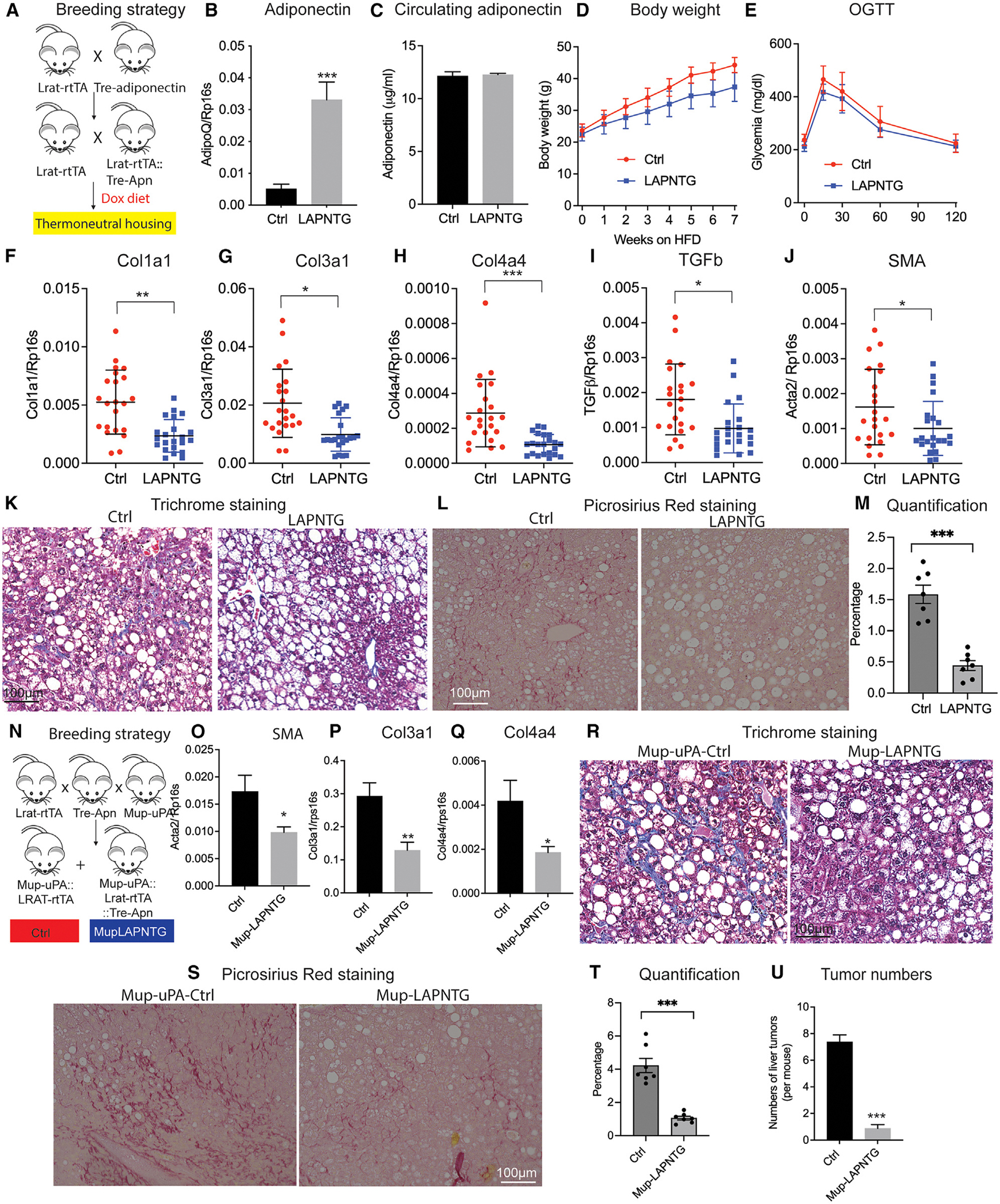
Overexpression of adiponectin in HSCs protects from thermoneutrality-induced liver fibrosis (A–K) Control (Lrat-rtTA) and LAPNTG (Lrat-rtTA+TRE-Adipoq) mice were housed under thermoneutral conditions and fed HFD+DOX to induce liver fibrosis. (A) Experimental setup. (B) Adiponectin mRNA expression in HSCs after 2 weeks’ doxycycline. (C) Circulating adiponectin levels after 2 weeks’ doxycycline (*n* = 5). (D) Body weight development. (E) OGTT after 8 weeks (*n* = 5–8). (F–J) Liver mRNA expression of (F) Col1a1, (G) Col3a1, (H) Col4a4, (I) TGF-β, and (J) smooth muscle actin after 1 year under thermoneutral conditions. (K) Liver trichrome staining. Scale bar represents 100 μm. (L) Picrosirius red staining. Scale bar represents 100 μm. (M) Quantification of fibrotic area. (N–U) Control (MUP-uPA+Lrat-rTA) and MUPLAPNTG (MUP-uPA+Lrat-rtTA+TRE-Adipoq) mice were fed chow+DOX. (N) Experimental setup. (O–Q) Liver mRNA expression of (O) smooth muscle actin, (P) Col3a1, and (Q) Col4a4 after 20 weeks. (R) Liver trichrome staining after 20 weeks (*n* = 10). (S) Picrosirius red staining. (T) Quantification of fibrotic area. (U) Liver tumors after 20 weeks’ HFD plus Dox600 (*n* = 10). Data are displayed as mean ± SEM and analyzed by unpaired Student’s t test for all the figures: *p < 0.05, **p < 0.01, ***p < 0.001.

With the Lrat-rtTA mouse model validated, we next assessed whether local adiponectin expression in HSCs contributes to fibrosis progression during liver injury. We generated a model of HSC-specific adiponectin overexpression (LAPNTG) by crossing the Lrat-rtTA line with the TRE-adiponectin line (Figure 2A). Feeding LAPNTG mice a DOX-containing high-fat diet (HFD+DOX) resulted in a significant increase in the expression of adiponectin in HSCs while circulating adiponectin levels remained unchanged (Figures 2B and 2C). We observed no differences in body weight gain (though a trend toward weight reduction is apparent) or glucose tolerance between LAPNTG and control mice (Figures 2D and 2E), which suggests that the local overexpression of adiponectin in HSCs does not substantially alter systemic metabolism.

Thermoneutral housing exacerbates nonalcoholic fatty liver disease in mice and allows for sex-independent disease modeling by modulating hepatic inflammation and damage.^21,22^ We therefore housed LAPNTG mice under thermoneutral conditions for 1 year. Intriguingly, we found that LAPNTG mice are protected from liver fibrosis as reflected by a greatly reduced expression of fibrotic genes such as Col1a1, Col3a1, Col4a4, TGF-β, and SMA (Figures 2F–2J). In further support of the beneficial effects of local adiponectin overexpression, trichrome staining indicated a pronounced reduction in liver fibrosis in LAPNTG mice (Figure 2K). Moreover, we further performed picrosirius red staining and quantified the percentage of liver fibrosis area in the liver. We found that overexpression of adiponectin in LAPNTG mice visually reduced liver fibrosis and significantly reduced the percentage of the fibrotic area (Figures 2L and 2M). We furthermore detected a significant reduction of circulating leptin, aspartate aminotransferase, and alanine aminotransferase levels in LAPNTG mice whereas cholesterol and triacylglycerol levels were comparable to those of control mice (Figures S3A–S3E). This indicates that increasing adiponectin expression in HSCs reduces liver injury and augments liver function to an extent sufficient to prevent thermoneutrality-induced liver fibrosis.

As an alternative approach to model liver fibrosis, we crossed the LAPNTG line with the MUP-uPA line to generate MUPLAPNTG mice (Figure 2N). MUP-uPA mice suffer from constitutively elevated endoplasmic reticulum (ER) stress in the liver and are a widely used model of steatosis-driven liver fibrosis and HCC development.^23,24^ Feeding MUPLAPNTG mice HFD+DOX for 20 weeks, we observed a significant reduction in the liver expression of fibrotic genes including SMA, Col3a1, and Col4a4 (Figures 2O–2Q). Furthermore, trichrome and picrosirius red staining of the liver indicated far less collagen deposition in the liver of MUPLAPNTG mice, indicative of reduced fibrotic change (Figures 2R and 2S). In addition, the quantification of the fibrotic area in the livers of Mup-LAPNTG mice showed a great reduction, indicating the protective effects of adiponectin on liver fibrosis (Figure 2T). Interestingly, as liver fibrosis is a major driver for the development of liver tumors, we wondered whether prevention of liver fibrosis in Mup-LAPNTG mice would have any protective effects on the progression to HCC. We found that MUPLAPNTG mice also displayed a dramatically reduced burden of liver tumors (Figure 2U). Taken together, these results suggest that local overexpression of adiponectin in HSCs not only protects from liver fibrosis but also delays the progression toward HCC.

### Deletion of adiponectin in HSCs exacerbates thermoneutrality-induced liver fibrosis

**Figure 3.**
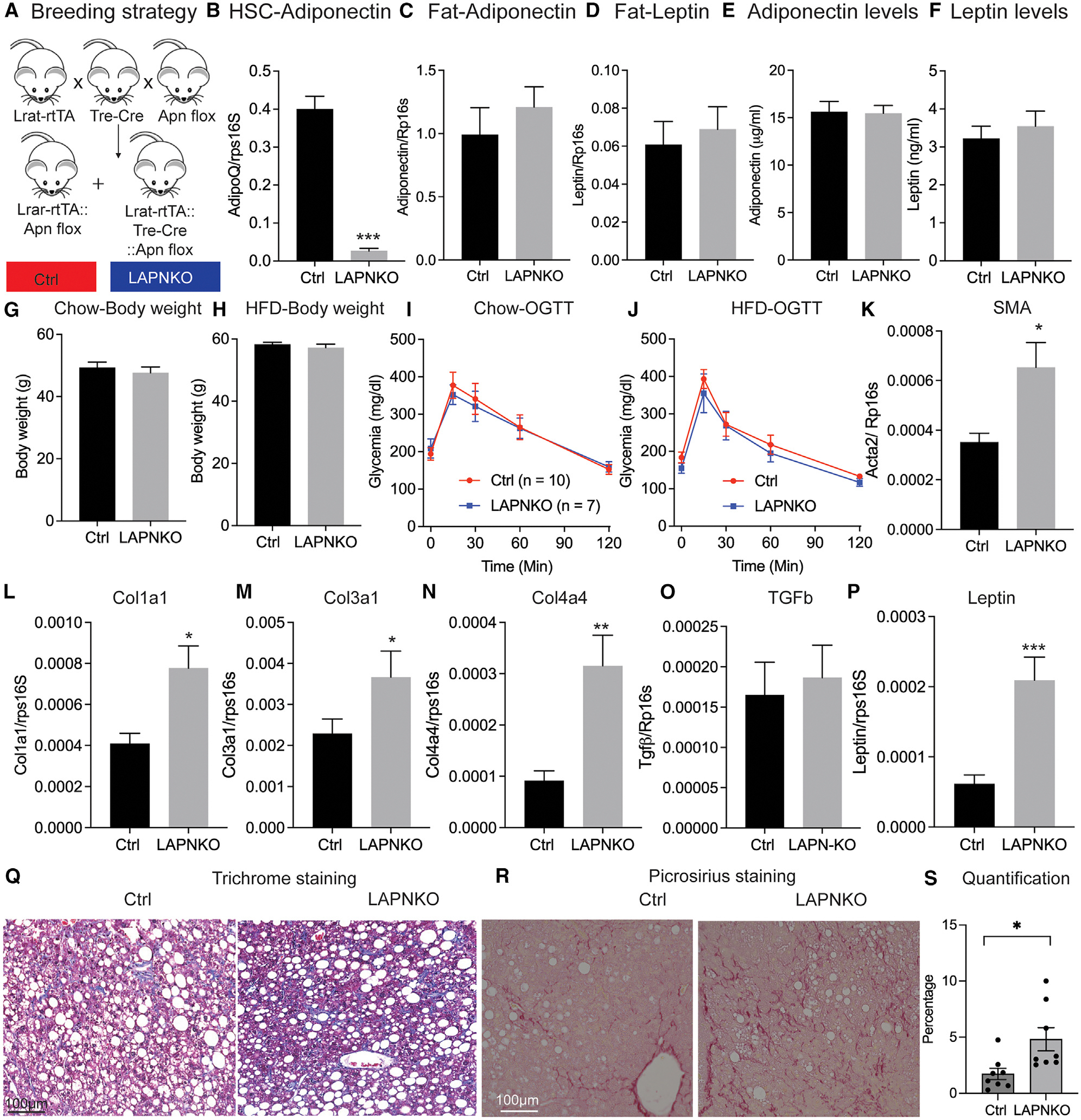
Deletion of adiponectin in HSCs exacerbates thermoneutrality-induced liver fibrosis Control (Lrat-rtTA+Adipoq flox/flox) and LAPNKO (Lrat-rtTA+TRE-Cre+Adipoq flox/flox) mice were housed under thermoneutral conditions and fed either chow+DOX or HFD+DOX to induce liver fibrosis. (A) Experimental setup. (B–D) mRNA expression of (B) adiponectin in HSCs, (C) adiponectin in subcutaneous adipose tissue, and (D) leptin in subcutaneous adipose tissue after 10 weeks (*n* = 8). (E and F) Circulating levels of (E) adiponectin and (F) leptin after 10 weeks. (G) Body weight of chow+DOX-fed mice after 10 weeks (*n* = 8). (H) Body weight of HFD+DOX-fed mice after 20 weeks (*n* = 10). (I) OGTT of chow+DOX-fed mice after 8 weeks (*n* = 7–10). (J) OGTT of HFD+DOX-fed mice after 8 weeks (*n* = 10). (K–P) Liver mRNA expression of (K) smooth muscle actin, (L) Col1a1, (M) Col3a1, (N) Col4a4, (O) TGF-β, and (P) leptin expression in HFD+DOX-fed mice after 20 weeks (*n* = 10). (Q) Liver trichrome staining of HFD+DOX-fed mice after 20 weeks (*n* = 10). Scale bar represents 100 μm. (R) Picrosirius red staining. Scale bar represents 100 μm. (S) Quantification of fibrotic area. Data are displayed as mean ± SEM and analyzed by unpaired Student’s t test for all the panels: *p < 0.05, **p < 0.01, ***p < 0.001.

As overexpression of adiponectin in HSCs protects mice from liver fibrosis, we wanted to test whether the loss of adiponectin in HSCs would worsen liver fibrosis, similar to what we observed *in vitro*. To this end, we crossed the Lrat-rtTA line with TRE-Cre and adiponectin floxed lines to generate LAPNKO mice (Figure 3A). Feeding LAPNKO mice HFD+DOX effectively disrupted the adiponectin gene in HSCs as indicated by a significant reduction in adiponectin expression in these cells (Figure 3B). Importantly, this deletion of adiponectin in HSCs neither affected adiponectin and leptin expression in subcutaneous adipose tissue nor did it have any impact on circulating adiponectin and leptin levels (Figures 3C–3F). This demonstrates that HSCs are not a significant source of circulating adiponectin. We observed only marginal differences in body weight development and no differences in the glucose tolerance of LAPNKO, either on chow+DOX or HFD+DOX (Figures 3G–3J), supporting our previous conclusions that HSC-derived adiponectin plays only a minor role in the regulation of systemic metabolism.

To promote the development of liver fibrosis, we housed LAPNKO mice under thermoneutral conditions and challenged them with HFD+DOX for 1 year, analogous to our experiments in LAPNTG mice. Doing so, we found that the deletion of adiponectin in HSCs accelerates the development of liver fibrosis as reflected by significant increases in SMA, Col1a1, Col3a1, Col4a4, and leptin, but not TGF-β expression in the liver (Figures 3K–3P). In line with the gene expression data, liver trichrome staining revealed a widespread increase in collagen deposition in LAPNKO (Figure 3Q). Moreover, we further performed picrosirius red staining and quantified the percentage of liver fibrosis area in the liver, and we found that deletion of adiponectin in LAPNKO mice significantly accelerated liver fibrosis and the percentage of fibrotic area (Figures 3R and 3S). These observations highlight HSC-derived adiponectin as an important endogenous regulator of liver fibrosis.

### An adiponectin-PPARγ axis mediates the beneficial effects of adiponectin in liver fibrosis

**Figure 4.**
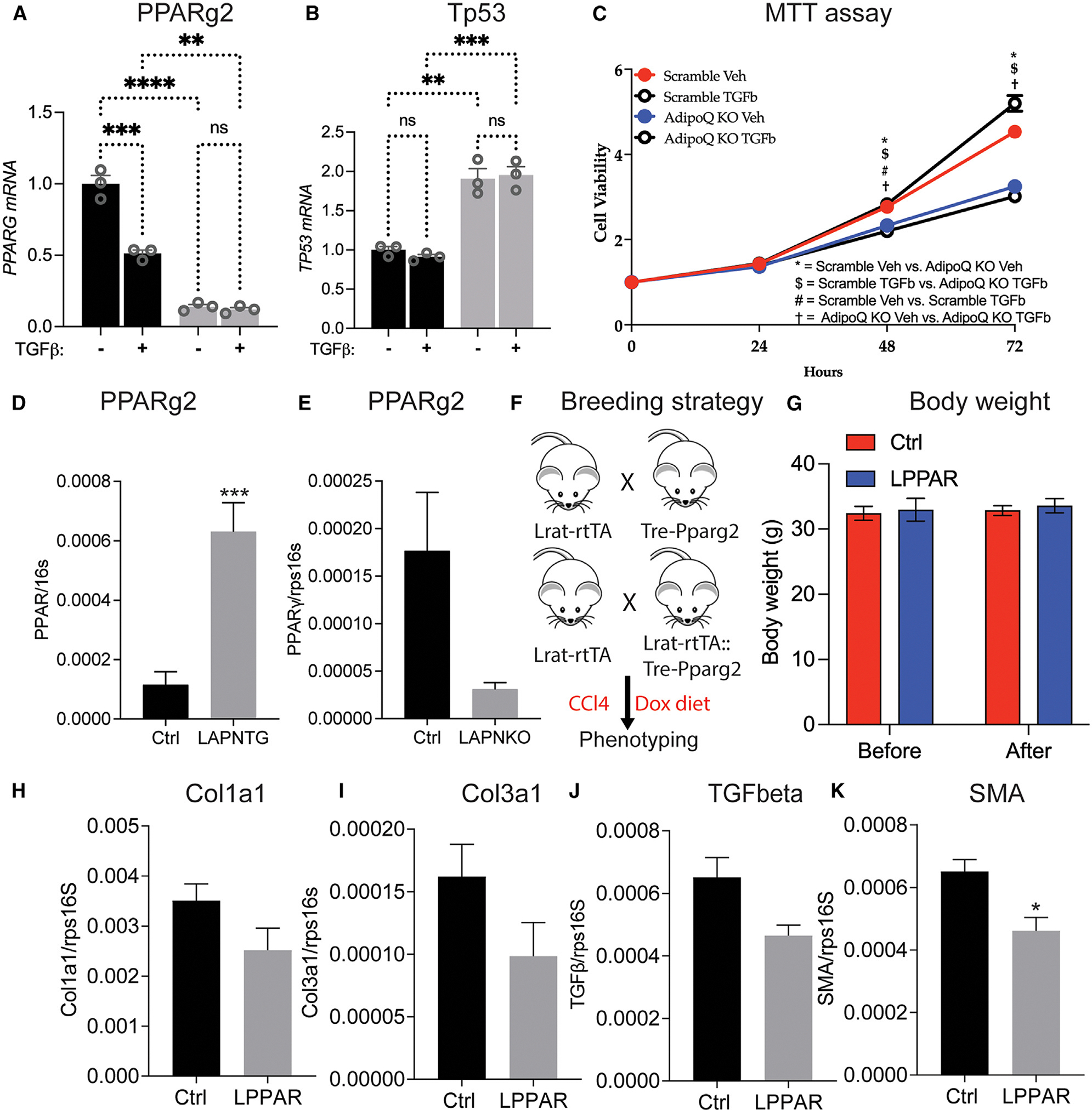
Overexpression of PPARγ in HSCs protects from CCl_4_-induced liver fibrosis (A–C) Ctrl and LX2KO cells were cultured in the presence and absence of TGF-β. Quantification of (A) PPARγ2 and (B) TP53 and (C) MTT assay, to quantify cell proliferation, was performed. (D) HSC PPARγ2 mRNA expression in LAPNTG mice fed HFD+DOX600 for 10 weeks (*n* = 5). (E) HSC PPARγ2 mRNA expression in LAPNKO mice fed HFD+DOX600 for 10 weeks (*n* = 5). (F–K) Control (Lrat-rtTA) and LPPARG2 (Lrat-rtTA+TRE-PPARgγ2) mice were fed chow+DOX and then injected twice weekly with CCl_4_ for another 8 weeks to induce liver fibrosis. (F) Experimental setup. (G) Body weight before and after CCl_4_ injection. (H–K) Liver mRNA expression of (H) Col1a1, (I) Col3a1, (J) TGF-β, and (K) smooth muscle actin (*n* = 5). Data are displayed as mean ± SEM and analyzed by unpaired Student’s t test for all the panels, except (C), which was analyzed by two-way ANOVA: *p < 0.05, **p < 0.01, ***p < 0.001.

Previously, we and others have shown that some of the beneficial effects of adiponectin are mediated in a PPARγ-dependent manner.^25^ To investigate the potential involvement of PPARγ in the beneficial effects of local adiponectin expression on liver fibrosis, we first determined PPARγ expression levels in the Ctrl and LX2KO cell line. We found that the reduction of adiponectin in LX2 cells dramatically reduced the expression of PPARγ (Figure 4A), suggesting the possible involvement of PPARγ in adiponectin-mediated suppression of liver fibrosis. As PPARγ is a master regulator of adipogenesis and may play a pivotal role in maintaining the HSCs in their quiescent state, we examined the expression of Tp53 and proliferation of LX2 cells in the absence and presence of TGF-β. In agreement with the role of PPARγ in adipogenesis, the deletion of adiponectin in LX2 cells significantly reduces the proliferation rate of the cells (Figures 4B and 4C). To further support the role of adiponectin in regulating PPARγ expression, we determined PPARγ expression in HSCs of LAPNTG and LAPNKO mice. Doing so, we found that adiponectin overexpression in stellate cells significantly increases HSC PPARγ levels. In contrast, stellate cell-specific adiponectin deletion significantly decreases PPARγ (Figures 4D and 4E). These observations suggest a mechanistic connection between adiponectin signaling and PPARγ expression in HSCs.

To further examine the role of HSC PPARγ expression in liver fibrosis, we crossed the Lrat-rtTA line with the TRE-PPARγ2 line to generate LPPARG2 mice (Figure 4F). We fed these mice chow+DOX and induced liver fibrosis by CCl_4_ injection. While we detected no effects of HSC PPARγ2 overexpression on the body weights of LPPARG2 mice (Figure 4G), we observed a moderate reduction in the liver expression of fibrotic genes Col1a1, Col3a1, TGFeta, and SMA (Figures 4H–4K). This may reflect a direct involvement of PPARγ in mediating the beneficial effects of adiponectin on liver fibrosis, as overexpression of PPARγ2 alone at least partially recapitulated the phenotype of adiponectin overexpression in HSCs. However, as PPARγ is a ligand-driven transcription factor, adiponectin may additionally further increase PPARγ ligand availability.

## DISCUSSION

Using both *in vitro* as well as *in vivo* transgenic mouse models, this study has yielded a number of important insights: first, our inducible HSC elimination model confirms that HSCs are the main cell type contributing to fibrosis progression during liver injury; second, our cell culture experiments demonstrate that HSCs express relevant amounts of adiponectin and that HSC activation is associated with a dramatic reduction in adiponectin expression; third, our inducible HSC adiponectin overexpression and elimination models reveal that *local* adiponectin expression in these cells shapes the development of liver fibrosis; fourth, we implicate PPARγ2 as a mediator of at least part of the beneficial effects of adiponectin signaling in HSCs. Based on these observations, we propose that the stellate cell-specific adiponectin-PPARγ axis in HSCs is a crucial regulator of liver fibrosis.

The beneficial effects of HSC adiponectin signaling on liver fibrosis are well documented, but it is generally believed that it is circulating adiponectin that keeps HSCs in their quiescent state. Consistent with the notion that a receptor-mediated signaling process conveys a signal derived from circulating adiponectin, we have shown that AdipoRon, a potent AdipoR agonist, exerts protective effects in liver fibrosis models.^26^ The contribution of local adiponectin expression in HSCs has, however, remained unaddressed by previous studies. Herein, we not only confirm that HSCs express significant amounts of adiponectin but also delineate its important contribution to the prevention of fibrotic changes in the liver. Even though the deletion of adiponectin exclusively in HSCs has no impact on the circulating adiponectin pool, it is sufficient to dramatically accelerate liver fibrosis progression in different mouse models of liver injury. Circulating adiponectin is unable to compensate for the local loss of adiponectin with the HSCs. In contrast, overexpressing adiponectin in HSCs potently protects mice from liver fibrosis. Yet, the mechanisms underlying these potent effects of HSC-derived adiponectin on liver fibrosis have not been fully elucidated. While autocrine signaling is a likely contributor, we are currently investigating alternative mechanisms of adiponectin action in HSCs as well. These include strictly intracellular actions of adiponectin as a local factor in the ER lumen, cytoplasm, or even nucleus. We, for instance, observed that instead of just being translocated into the ER and secreted from the cell, adiponectin is partially retained in the cytoplasm where it may directly interact with different factors, including PPARγ (*Onodera and Scherer*, manuscript under review).

Our observations regarding the relationship between adiponectin and PPARγ shed new light on local adiponectin action within target cells. Previously, many reports indicated strong correlations between the regulation of adiponectin and PPARγ expression. We and others have shown that activation of PPARγ is associated with increased adiponectin secretion from adipocytes. The synthetic PPARγ agonist rosiglitazone, for example, strongly stimulates the secretion of adiponectin from adipocytes and elevates its circulating levels in a PPARγ-dependent manner.^27^,^28^ There are however also many observations that adiponectin expression and signaling may directly impact PPARγ activity. As such, the inducible deletion of adiponectin in adipocytes is tied to a lower PPARγ levels. Such observations reflect a complex relationship between adiponectin and PPARγ expression and activity. How exactly adiponectin expression and signaling affect PPARγ activity remains to be determined though.

As a part of this study, we also delineated the contributions of HSCs and HSC-derived adiponectin to energy and nutrient homeostasis. Despite their importance for local collagen deposition during liver injury, it seems that HSCs exert relatively minor effects on systemic parameters. As such, the near total elimination of HSCs did not have any relevant impact on either body weight development or systemic glucose homeostasis, and neither did the overexpression nor elimination of adiponectin in HSCs. Additionally, adiponectin is known for its potent role in suppressing inflammation. Our results found that the deletion of adiponectin in LX2 cells potently increases inflammation markers, indicative of the crucial role of adiponectin in HSCs. However, we do not know whether the anti-inflammatory and anti-fibrotic activities of adiponectin in vivo rely on each other or constitute separate events. Beyond our finding that adiponectin expression in HSCs alleviates liver fibrosis, we also uncovered a connection of HSC-derived adiponectin on liver steatosis and HCC development, as previously postulated.^24^ Mice fed with an MCD diet develop severe liver steatosis, as reflected by massive lipid droplet accumulation in the liver. However, the elimination of HSCs in LATTAC mice not only prevents the development of liver fibrosis but also liver steatosis, which is highly interesting. We believe that liver steatosis and liver fibrosis are interrelated. Chronic liver steatosis can lead to liver fibrosis, while liver fibrosis further impairs liver function and exacerbates liver steatosis. Thus, preventing liver fibrosis by eliminating HSCs can improve liver function and prevent lipid droplet accumulation in the liver, resulting in reduced liver steatosis. Moreover, while overexpression of adiponectin in HSCs in the MUP-uPA model did not prevent the occurrence of severe liver steatosis, there was a striking reduction in the tumor burden of these mice. This is consistent with our observations in a previous aging study in which we found that adiponectin-deficient mice not only develop severe liver fibrosis but also display an accelerated progression toward HCC. This supports the widely held notion that liver fibrosis is a prerequisite step toward progression to HCC.

In summary, HSC-derived adiponectin plays a pivotal role in maintaining HSCs in a quiescent state, which may involve an elevation of PPARγ levels in HSCs. Deficient adiponectin expression in HSCs consequently promotes their activation, initiates fibrotic gene expression, and drives liver fibrosis. The adiponectin-PPARγ axis in HSCs constitutes a potential therapeutic target to prevent or reverse liver fibrosis and contain HCC progression in patients.

### Limitations of the study

Here, we provide evidence demonstrating that HSC-derived adiponectin regulates liver fibrosis via PPARγ, and overexpression of PPARγ can partially recapitulate the phenotypes of adiponectin overexpression in HSCs. Our studies do not address how exactly locally produced adiponectin exerts its antifibrotic actions and how it leads to enhanced transcription and/or stabilization of PPARγ. Is there a pool of adiponectin that does not enter the secretory pathway but rather remains in the cytoplasm where it can partner with other proteins, potentially even directly with PPARγ, to exert its effect through an “intracrine” effect? Or is locally produced adiponectin released into the cellular microenvironment and taken back up through an autocrine mechanism? In terms of progression from healthy liver to metabolic dysfunction-associated steatotic liver disease (MASLD) to metabolic dysfunction-associated steatohepatitis, we do not know when exactly the downregulation of adiponectin occurs and whether this downregulation merely exacerbates disease progression or whether it serves as a disease initiator. If so, what are the most proximal events that lead to the reduction in local HSC production?

## RESOURCE AVAILABILITY

### Lead contact

Requests for further information, resources, and reagents should be directed to and will be fulfilled by the lead contact, Dr. Philipp E. Scherer (philipp.scherer@utsouthwestern.edu).

### Materials availability

Mouse lines generated in this study can be obtained from UT Southwestern Medical Center from the lead contact, Dr. Philipp E. Scherer.

### Data and code availability

All the data reported will be shared by the lead contact upon request.

This paper does not report any original code.

Any additional information regarding the data can be requested from the lead contact.

## STAR★METHODSs

### EXPERIMENTAL MODEL AND STUDY PARTICIPANT DETAILS

All animal experimental protocols have been approved by the Institutional Animal Care and Use Committee of University of Texas Southwestern Medical Center at Dallas. All mice were housed under standard laboratory conditions (12 h on/off; lights on at 7:00 a.m.) and temperature-controlled environment with food and water available *ad libitum*. Mice were fed a standard chow-diet (number 5058, LabDiet, St. Louis, MO), a Doxcycline (DOX)-chow diet (600 mg/kg Dox; BioServ, Frenchtown, NJ) or HFD with HFD-DOX600 (600 mg/kg Dox) (Made by BioServ upon special request, Frenchtown, NJ) for various periods as indicated in the figure legends. All experiments were initiated at approximately 7 or 8 weeks of age, unless indicated otherwise. Investigators were not blinded to treatment groups during studies. In order to ensure the accuracy of each experiment, we have repeated the mouse phenotyping studies with minimally two separate cohorts with more than 5 mice in each group.

As this study involved a large number of different mouse lines, we list the detailed information for all mouse lines with respect to mouse background, breeding strategy and genotyping assays that were used in this study:

#### Lrat-rtTA mice:

This mouse line was generated by Dr. Scherer’s lab. A transgene construct containing a *lecithin retinol acyltransferase* (*Lrat*) promoter-driven rtTA was linearized, purified, and injected into mouse zygotes by the UTSW Transgenic Technology Core. F0 founders delivered by foster mothers were screened for transgene integration using PCR on genomic DNA. Transgene-positive F0 founders were propagated and a line displaying rtTA mRNA expression specifically in HSCs but not in other tissues was identified. Genotyping primers were listed in the STAR Methods table.

#### TRE-GFP mice:

This mouse line was obtained from The Jackson Laboratory. We crossed this mouse line with Lrat-rtTA mice to determine the specificity of lrat-promoter in hepatic stellate cells.

#### TRE-Adipoq mice:

This mouse line was described and validated by us previously.^19,29^ In this study, we have crossed this mouse line with lrat-rtTA mice to determine the local effect of adiponectin in liver fibrosis under various conditions.

#### Adipoq floxed mice:

This mouse line was described and validated by us previously.^31^ In this study, we have used this mouse line to determine the local effect of adiponectin in liver fibrosis.

#### TRE-Cre mice:

This mouse line was obtained from The Jackson Laboratory (#006234). In combination with Lrat-rtTA mice, This mice has been used to specifically drive Cre expression in hepatic stellate cells.

#### MUP-uPA mice:

This mouse line was a kind gift from Dr. Michael Karin.^24^ These mice develop liver steatosis, liver fibrosis and progress to hepatocellular carcinoma when fed a high-fat diet for 20 weeks or more.

#### TRE-PPARγ2 mice:

This mouse line was described and validated by us previously.^30^ In this study, we have observed an increased expressed PPARγ2 in HSC-specific adiponectin transgenic mice. And we used this mouse line to specifically overexpress PPARγ2 to mimics the effects of adiponectin overexpression.

### METHOD DETAILS

#### Glucose tolerance test

Glucose tolerance tests (GTT) was performed as previously described.^32^ In brief, for GTT, mice were fasted 4–6 h in the morning, and the mice were received 2g glucose per kg body weight at the volume calculated based on 10ul/g body weight and blood glucose was measured by glucose meter as indicated time points (0, 15, 30, 60, 120 min), shown in different figures.

##### Thermoneutrality-induced liver fibrosis:

Housing mice under thermoneutrality has been reported to accelerate the development of liver fibrosis. Here, mice were housed under thermoneutral conditions (30°C) and while also being fed a high-fat diet.

##### CCl_4_-induced liver fibrosis:

Mice were fed Chow+DOX and then injected intraperitoneally with either CCl_4_ (0.2 mL/kg of body weight; Sigma-Aldrich, #289116) or corn oil (Sigma-Aldrich, #C8267) twice weekly (every Monday and Thursday afteronoon) for 8 consecutive weeks.

##### Insulin, leptin, and adiponectin measurements:

Circulating insulin and leptin levels were measured using an ELISA kit from Crystal Chem (Cat #90030), adiponectin levels were measured using an ELISA kit from ThermoFisher (Catalog #KMP0041).

##### LX-2 cell culture:

The human hepatic stellate cell line LX-2 was obtained from Millipore Sigma (#SCC064) and cultured according to the manufacturer’s recommendations. All experiments were performed between passages 4 to 6. For experiments, LX-2 cells were grown to 75% confluence and then starved overnight in serum-free medium. The starved cells were then incubated in the treated with 20 ng/mL TGFβ (Millipore-Sigma, Cat: #H8541) and/or 1μM AdipoRon (Millipore-Sigma, Cat: #SML0998) or vehicle for 24 h.

#### ADIPOQ CRISPR knockout and validation

LX2 HSC cells were used to generate the CRISPR knockout of the *ADIPOQ* gene using dual gRNAs expressed by a plasmid from VectorBuilder (Chicago, IL). The vector has a green fluorescent protein (GFP) fused with a puromycin resistance marker, which was used for selection. The plasmid was co-transfected with another plasmid for the expression of Cas9 in the LX2 cells. Twenty-four hours after transfection, GFP expression in the cells appeared, and puromycin selection was performed, establishing a stable *ADIPOQ* knockout HSC cell line. To validate the *ADIPOQ* CRISPR knockout in these cells, we extracted genomic DNA (gDNA) and performed PCR with *ADIPOQ* gDNA genotyping using forward primer CTATTAGCTCTGCCCGGTCA and reverse primer GGTCGTGTCACTGTACTCCA, which are near the CRISPR cut sites. We also validated the mRNA level by quantitative Real-Time PCR using primers recognizing the mRNA area within the CRISPR cut site.

#### Proliferation assay

The HSCs were seeded in 24-well culture plates (one plate for a time point) at a confluency of 5000 cells per well and were then incubated at 37°C and 5% CO2. The 5 ng/mL Tgfβ treatment starts on the next day. Every 24 h of the treatment, a plate was treated with 100μL/well of MTT (3-(4,5-Dimethylthiazol-2-yl)-2,5-Diphenyltetrazolium Bromide) solution and incubated in the dark within the CO2 incubator for 4 h. Afterward, the medium was discarded, and the formazan crystals produced by the cells were solubilized in 300μL/well of DMSO. A 100μL aliquot of this solution was then transferred to a 96-well plate for absorbance measurement at 570nm using a Varioskan LUX multiwell plate reader from ThermoFisher Scientific, USA.

##### Primary hepatic stellate cell isolation and culture:

Mouse hepatic stellate cells were isolated from mouse livers using a combination of enzymatic digestion and density gradient centrifuge.^33^ The liver was perfused with EGTA solution and pronase (14mg per mouse) and collagenase (3.7 Unit per mouse). The density gradient was used Nycodenz solution with 4.94 g of Nycodenz in 15 mL of GBSS/A solution (Sigma-Aldrich cat. #G9779). After gradient centrigufe, the puried hepatic stellate cells were located in the top layer. HSC cells were then cultured in DMEM (ThermoFisher, CAT10565042) supplemented 10% fetal bovine serum (Millipore-Sigma, Cat #F2442).

##### RT-qPCR:

RNA was extracted from fresh or frozen tissues by homogenization in TRIzol Reagent (Thermo Fisher Scientific) as previously described.^34^ A total of 500 ng RNA was reverse transcribed using the iScript cDNA Synthesis Kit (Bio-Rad). RT-qPCR was performed on a QuantStudio 6 system (Thermo Fisher Scientific) using PowerUp SYBR Green Master Mix (Thermo Fisher Scientific). Most primer sequences were obtained from Harvard PrimerBank (https://pga.mgh.harvard.edu/primerbank/). Relative expression levels were calculated using the comparative threshold cycle method with normalization to the housekeeping gene Rps16.

##### Histology and immunohistochemistry:

Tissues were collected, fixed overnight in 10% PBS-buffered formalin, and then transferred to 50% ethanol for long-term storage. Fixed tissues were processed by the UTSW Molecular Pathology Core. Trichrome staining and Picrosirius Red staining were performed in the UTSW Molecular Pathology Core. To quantify the percentage of fibrotic area in liver sections, we have randomly taken 10 sections of liver per mouse and used ImageJ to calculate the area.

### QUANTIFICATION AND STATISTICAL ANALYSIS

Data are displayed as mean ± SEM. All statistical analyses were performed using Prism (version 9.5.1; GraphPad). Student’s t tests were used for comparisons between two groups and one-way or two-way ANOVAs were used for comparisons between more than two groups. The statistival details of experiments can be found in the figure legends with exact n number used in each study. A *p* value <0.05 was considered statistically significant and significance levels were demontreted as *p < 0.05, **p < 0.001, and ***p < 0.0001.

## Supplementary Material

1

## Figures and Tables

**KEY RESOURCES TABLE:** 

REAGENT or RESOURCE	SOURCE	IDENTIFIER

Antibodies		

Rabbit anti-adiponectin antibody	This paper	Scherer lab
Chicken anti-GFP antibody	Abcam	Cat# ab13970; RRID: AB_300798

Chemicals, peptides, and recombinant proteins		

Glucose	GIBCO	Cat# 15023-021
Insulin	Lilly	Cat# A10008415
Collagenase D	Roche	Cat# 11088882001
Collagenase B	Roche	Cat# 11088831001
TGFβ	Millipore-Sigma	Cat# H8541
AdipoRon	Millipore-Sigma	Cat# SML0998
CCl_4_	Millipore-Sigma	Cat# 289116
Corn oil	Millipore-Sigma	Cat# C8267
DMSO	Millipore-Sigma	Cat# D8418
MTT	Millipore-Sigma	Cat# 475989
DMEM	ThermoFisher	Cat# 10565042
FBS	Millipore-Sigma	Cat# F2442
BSA	Sigma-Aldrich	Cat# A8806

Critical commercial assays		

Mouse leptin ELISA kit	Crystal Chem	Cat# 90030
LabAssay^™^ Triglyceride	Wako	Cat# 632–50991
LabAssay^™^ Cholesterol	Wako	Cat# 635–50981
iScript cDNA synthesis kit	BIO-RAD	Cat# 170–8891
SYBR Green Master Mix	Applied Biosystems	Cat# A25778

Experimental models: organisms/strains		

mouse: WT C57BL6/J	Jackson Laboratory	JAX. 000664
Mouse: Tre-Cre	The Jackson Laboratory	JAX: 006234; RRID: IMSR_JAX:006234
Mouse: Tre-GFP	Jackson Laboratory	RRID: IMSR_JAX:018913
Mouse: Tre-Cas8	Generated in-house	N/A
Mouse: Lrat-rtTA	Generated in-house	N/A
Mouse: Tre-Adiponectin	Rutkowski et al.^19,29^	N/A
Mouse: Mup-uPA	Nakagawa et al.^24^	N/A
Mouse: Tre-PPARγ2	Shao et al.^30^	N/A

Oligonucleotides		

Genotyping forward primer for Lrat-rtTA: 5’- atctcttgctctcctggctct -3’	Sigma	N/A
Genotyping reverse primer for Lrat-rtTA: 5’- agcttttgagcgagtttcctt -3’	Sigma	N/A
Genotyping forward primer for Tre-AdopoQ: 5’- GACCACAATGGACTCTATG -3’	Sigma	N/A
Genotyping reverse primer for Tre-AdipoQ: 5’- CAAGGGACATCTTCCCATTC -3’	Sigma	N/A
Genotyping forward primer for AdipoQ floxed: 5’- GGTGGCTCACAACCATTCATAA -3’	Sigma	N/A
Genotyping reverse primer for AdipoQ floxed: 5’- CATACTCGCCTCTCCCAGAG -3’	Sigma	N/A
Genotyping forward primer for Tre-Cre mice: 5’- GATTTCGACCAGGTTCGTTC -3’	Sigma	N/A
Genotyping reverse primer for Tre-Cre mice: 5’- GCTAACCAGCGTTTTCGTTC -3’	Sigma	N/A
Genotyping forward primer for Tre-Cas8 mice: 5’- TGGTTTCTTTGGGCTAGAGG -3’	Sigma	N/A
Genotyping reverse primer for Tre-Cas8 mice: 5’- ACCTTCCGCTTCTTCTTTGG -3’	Sigma	N/A
MusTfgb qPCR Forward Primer: 5’- TTATTGAGCACCTTGGGCACT -3’	Sigma	N/A
Mus Tgfb qPCR reverse Primer: 5’- TGGGCTTGTTTCCTCACCTT -3’	Sigma	N/A
Mus Leptin qPCR Forward Primer: 5’- GAGACCCCTGTGTCGGTTC-3’	Sigma	N/A
Mus Leptin qPCR Reverse Primer: 5’- CTGCGTGTGTGAAATGTCATTG-3’	Sigma	N/A
Mus Adiponectin qPCR Forward Primer: 5’- TGTTCCTCTTAATCCTGCCCA-3’	Sigma	N/A
Mus Adiponectin qPCR Reverse Primer: 5’- CCAACCTGCACAAGTTCCCTT-3’	Sigma	N/A
Mus Foxsl qPCR Forward Primer: 5’- ACAACTGAGCCAACCAAGCC -3’	Sigma	N/A
Mus Foxsl qPCR Reverse Primer: 5’- GCGAATCGGCCCATGATGTA -3’	Sigma	N/A
Mus Acta2 qPCR Forward Primer: 5’- ACTGCCTTGGTGTGTGACAA -3’	Sigma	N/A
Mus Acta2 qPCR Reverse Primer: 5’- CACCATCACCCCCTGATGTC -3’	Sigma	N/A
Mus TNFα qPCR Forward Primer: 5’- GACGTGGAACTGGCAGAAGAG -3’	Sigma	N/A
Mus TNFα qPCR Reverse Primer: 5’- TTGGTGGTTTGTGAGTGTGAG -3’	Sigma	N/A
Mus Pparg qPCR Forward Primer: 5’- ACAATGCTGGCCTCCTTGAT -3’	Sigma	N/A
Mus Pparg qPCR Reverse Primer: 5’- AGGCTTTCGCAGGCTCTTTA -3’	Sigma	N/A
MusCollal qPCR Forward Primer: 5’- CCTGGAATGAAGGGACACAGA -3’	Sigma	N/A
Mus Collai qPCR Reverse Primer: 5’- AGCACCATCATTTCCACGA -3’	Sigma	N/A
Mus Serpinel qPCR Forward Primer: 5’- GACCGCAACGTGGTTTTCTC -3’	Sigma	N/A
Mus Serpinel qPCR Reverse Primer: 5’- GCCATGCCCTTGTCATCAAT -3’	Sigma	N/A
Mus IL-1β qPCR Forward Primer: 5’- TCGCCAGTGAAATGATGGCT -3’	Sigma	N/A
Mus IL-1β qPCR Reverse Primer: 5’- GGTCGGAGATTCGTAGCTGG -3’	Sigma	N/A
Mus Ccl2 qPCR Forward Primer: 5’- TAGCAGCCACCTTCATTCCC -3’	Sigma	N/A
Mus Ccl2 qPCR Reverse Primer: 5’- ATAACAGCAGGTGACTGGGG -3’	Sigma	N/A
Mus IL-6 qPCR Forward Primer: 5’- AGCCCACCGGGAACGA -3’	Sigma	N/A
Mus IL-6 qPCR Reverse Primer: 5’- CCGAAGGCGCTTGTGGAGA -3’	Sigma	N/A
Mus Ifng qPCR Forward Primer: 5’- TGCAATCTGAGCCAGTGCTT -3’	Sigma	N/A
Mus Ifng qPCR Reverse Primer: 5’- GCACCAGGCATGAAATCTCC -3’	Sigma	N/A
Mus Adgrel qPCR Forward Primer: 5’- AATCAGGCGTTTCTGCTCCA -3’	Sigma	N/A
Mus Adgrel qPCR Reverse Primer: 5’- TGGAGGGGCATGCTTAGAAC -3’	Sigma	N/A
Mus Tp53 qPCR Forward Primer: 5’- ACCTATGGAAACTACTTCCTGAAA -3’	Sigma	N/A
Mus Tp53 qPCR Reverse Primer: 5’- TCCGGGGACAGCATCAAATC -3’	Sigma	N/A
Mus Col4a4 qPCR Forward Primer: 5’- ATGAGGTGCTTTTTCAGATGGAC -3’	Sigma	N/A
Mus Col4a4 qPCR Reverse Primer: 5’- GGGGCCGCCATACTTCTTG -3’	Sigma	N/A

Software and algorithms		

Excel	Microsoft	N/A
Word	Microsoft	N/A
PowerPoint	Microsoft	N/A
Prism	GraphPad Software	GraphPad Software
Illustrator	Adobe	N/A
ImageJ	NIH	N/A

Other		

Keyence BZ-X700 Fluorescence Microscope	Keyence	N/A
Odyssey Infrared Imager	Li-Cor	N/A
60% HFD paste	BioServ	S1850
Normal Chow diet	Lab diet	5058
Doxycycline chow diet (600 mg/kg diet)	BioServ	N/A
Doxycycline High fat diet (600 mg/kg diet)	BioServ	N/A

## Highlights

- Lrat-rtTA mice were generated to target hepatic stellate cells specifically
- Overexpression of adiponectin in hepatic stellate cells prevents liver fibrosis
- Deletion of adiponectin in hepatic stellate cells accelerates liver fibrosis
- The effects of adiponectin in liver fibrosis are partially mediated via PPARγ

## References

[1] Kisseleva T, and Brenner D (2021). Molecular and cellular mechanisms of liver fibrosis and its regression. Nat. Rev. Gastroenterol. Hepatol. 18, 151–166. 10.1038/s41575-020-00372-7. 33128017

[2] Berumen J, Baglieri J, Kisseleva T, and Mekeel K (2021). Liver fibrosis: Pathophysiology and clinical implications. WIREs Mech. Dis. 13, e1499. 10.1002/wsbm.1499. 32713091 PMC9479486

[3] Scherer PE, Williams S, Fogliano M, Baldini G, and Lodish HF (1995). A novel serum protein similar to C1q, produced exclusively in adipocytes. J. Biol. Chem. 270, 26746–26749. 10.1074/jbc.270.45.26746. 7592907

[4] Berg AH, Combs TP, Du X, Brownlee M, and Scherer PE (2001). The adipocyte-secreted protein Acrp30 enhances hepatic insulin action. Nat. Med. 7, 947–953. 10.1038/90992. 11479628

[5] Zhao S, Kusminski CM, and Scherer PE (2021). Adiponectin, Leptin and Cardiovascular Disorders. Circ. Res. 128, 136–149. 10.1161/CIRCRESAHA.120.314458. 33411633 PMC7799441

[6] Kamada Y, Tamura S, Kiso S, Matsumoto H, Saji Y, Yoshida Y, Fukui K, Maeda N, Nishizawa H, Nagaretani H, (2003). Enhanced carbon tetrachloride-induced liver fibrosis in mice lacking adiponectin. Gastroenterology 125, 1796–1807. 10.1053/j.gastro.2003.08.029. 14724832

[7] Combs TP, Pajvani UB, Berg AH, Lin Y, Jelicks LA, Laplante M, Nawrocki AR, Rajala MW, Parlow AF, Cheeseboro L, (2004). A transgenic mouse with a deletion in the collagenous domain of adiponectin displays elevated circulating adiponectin and improved insulin sensitivity. Endocrinology 145, 367–383. 10.1210/en.2003-1068. 14576179

[8] Shafiei MS, Shetty S, Scherer PE, and Rockey DC (2011). Adiponectin regulation of stellate cell activation via PPARgamma-dependent and -independent mechanisms. Am. J. Pathol. 178, 2690–2699. 10.1016/j.ajpath.2011.02.035. 21641391 PMC3124230

[9] Kumar P, Smith T, Rahman K, Thorn NE, and Anania FA (2014). Adiponectin agonist ADP355 attenuates CCl4-induced liver fibrosis in mice. PLoS One 9, e110405. 10.1371/journal.pone.0110405. 25310107 PMC4195748

[10] Li N, Zhao S, Zhang Z, Zhu Y, Gliniak CM, Vishvanath L, An YA, Wang MY, Deng Y, Zhu Q, (2021). Adiponectin preserves metabolic fitness during aging. Elife 10, e65108. 10.7554/eLife.65108. 33904399 PMC8099426

[11] Dong Z, Su L, Esmaili S, Iseli TJ, Ramezani-Moghadam M, Hu L, Xu A, George J, and Wang J (2015). Adiponectin attenuates liver fibrosis by inducing nitric oxide production of hepatic stellate cells. J. Mol. Med. 93, 1327–1339. 10.1007/s00109-015-1313-z. 26153548

[12] Ramezani-Moghadam M, Wang J, Ho V, Iseli TJ, Alzahrani B, Xu A, Van der Poorten D, Qiao L, George J, and Hebbard L (2015). Adiponectin reduces hepatic stellate cell migration by promoting tissue inhibitor of metalloproteinase-1 (TIMP-1) secretion. J. Biol. Chem. 290, 5533–5542. 10.1074/jbc.M114.598011. 25575598 PMC4342468

[13] Pineiro R, Iglesias MJ, Gallego R, Raghay K, Eiras S, Rubio J, Dieguez C, Gualillo O, Gonzalez-Juanatey JR, and Lago F (2005). Adiponectin is synthesized and secreted by human and murine cardiomyocytes. FEBS Lett. 579, 5163–5169. 10.1016/j.febslet.2005.07.098. 16140297

[14] Shen YY, Charlesworth JA, Kelly JJ, Loi KW, and Peake PW (2007). Up-regulation of adiponectin, its isoforms and receptors in endstage kidney disease. Nephrol. Dial. Transplant. 22, 171–178. 10.1093/ndt/gfl552. 17005524

[15] Potter JJ, and Mezey E (2007). Acetaldehyde increases endogenous adiponectin and fibrogenesis in hepatic stellate cells but exogenous adiponectin inhibits fibrogenesis. Alcohol Clin. Exp. Res. 31, 2092–2100. 10.1111/j.1530-0277.2007.00529.x. 17949463

[16] Onodera T, Wang MY, Rutkowski JM, Deja S, Chen S, Balzer MS, Kim DS, Sun X, An YA, Field BC, (2023). Endogenous renal adiponectin drives gluconeogenesis through enhancing pyruvate and fatty acid utilization. Nat. Commun. 14, 6531. 10.1038/s41467-023-42188-4. 37848446 PMC10582045

[17] Pajvani UB, Trujillo ME, Combs TP, Iyengar P, Jelicks L, Roth KA, Kitsis RN, and Scherer PE (2005). Fat apoptosis through targeted activation of caspase 8: a new mouse model of inducible and reversible lipoatrophy. Nat. Med. 11, 797–803. 10.1038/nm1262. 15965483

[18] Wang ZV, Mu J, Schraw TD, Gautron L, Elmquist JK, Zhang BB, Brownlee M, and Scherer PE (2008). PANIC-ATTAC: a mouse model for inducible and reversible beta-cell ablation. Diabetes 57, 2137–2148. 10.2337/db07-1631. 18469203 PMC2494693

[19] Rutkowski JM, Wang ZV, Park ASD, Zhang J, Zhang D, Hu MC, Moe OW, Susztak K, and Scherer PE (2013). Adiponectin promotes functional recovery after podocyte ablation. J. Am. Soc. Nephrol. 24, 268–282. 10.1681/ASN.2012040414. 23334396 PMC3559480

[20] Tomita K, Tamiya G, Ando S, Ohsumi K, Chiyo T, Mizutani A, Kitamura N, Toda K, Kaneko T, Horie Y, (2006). Tumour necrosis factor alpha signalling through activation of Kupffer cells plays an essential role in liver fibrosis of non-alcoholic steatohepatitis in mice. Gut 55, 415–424. 10.1136/gut.2005.071118. 16174657 PMC1856073

[21] Giles DA, Moreno-Fernandez ME, Stankiewicz TE, Graspeuntner S, Cappelletti M, Wu D, Mukherjee R, Chan CC, Lawson MJ, Klarquist J, (2017). Thermoneutral housing exacerbates nonalcoholic fatty liver disease in mice and allows for sex-independent disease modeling. Nat. Med. 23, 829–838. 10.1038/nm.4346. 28604704 PMC5596511

[22] Oates JR, Sawada K, Giles DA, Alarcon PC, Damen MSMA, Szabo S, Stankiewicz TE, Moreno-Fernandez ME, and Divanovic S (2023). Thermoneutral housing shapes hepatic inflammation and damage in mouse models of non-alcoholic fatty liver disease. Front. Immunol. 14, 1095132. 10.3389/fimmu.2023.1095132. 36875069 PMC9982161

[23] Febbraio MA, Reibe S, Shalapour S, Ooi GJ, Watt MJ, and Karin M (2019). Preclinical Models for Studying NASH-Driven HCC: How Useful Are They? Cell Metab. 29, 18–26. 10.1016/j.cmet.2018.10.012. 30449681 PMC6326872

[24] Nakagawa H, Umemura A, Taniguchi K, Font-Burgada J, Dhar D, Ogata H, Zhong Z, Valasek MA, Seki E, Hidalgo J, (2014). ER stress cooperates with hypernutrition to trigger TNF-dependent spontaneous HCC development. Cancer Cell 26, 331–343. 10.1016/j.ccr.2014.07.001. 25132496 PMC4165611

[25] Rao JR, Keating DJ, Chen C, and Parkington HC (2012). Adiponectin increases insulin content and cell proliferation in MIN6 cells via PPARgamma-dependent and PPARgamma-independent mechanisms. Diabetes Obes. Metab 14, 983–989. 10.1111/j.1463-1326.2012.01626.x. 22594400

[26] Sha M, Gao Y, Deng C, Wan Y, Zhuang Y, Hu X, and Wang Y (2020). Therapeutic effects of AdipoRon on liver inflammation and fibrosis induced by CCl(4) in mice. Int. Immunopharmacol. 79, 106157. 10.1016/j.intimp.2019.106157. 31911372

[27] Yang WS, Jeng CY, Wu TJ, Tanaka S, Funahashi T, Matsuzawa Y, Wang JP, Chen CL, Tai TY, and Chuang LM (2002). Synthetic peroxisome proliferator-activated receptor-gamma agonist, rosiglitazone, increases plasma levels of adiponectin in type 2 diabetic patients. Diabetes Care 25, 376–380. 10.2337/diacare.25.2.376. 11815513

[28] Motoshima H, Wu X, Sinha MK, Hardy VE, Rosato EL, Barbot DJ, Rosato FE, and Goldstein BJ (2002). Differential regulation of adiponectin secretion from cultured human omental and subcutaneous adipocytes: effects of insulin and rosiglitazone. J. Clin. Endocrinol. Metab. 87, 5662–5667. 10.1210/jc.2002-020635. 12466369

[29] Rutkowski JM, Pastor J, Sun K, Park SK, Bobulescu IA, Chen CT, Moe OW, and Scherer PE (2017). Adiponectin alters renal calcium and phosphate excretion through regulation of klotho expression. Kidney Int. 91, 324–337. 10.1016/j.kint.2016.09.016. 27914707 PMC5237401

[30] Shao M, Vishvanath L, Busbuso NC, Hepler C, Shan B, Sharma AX, Chen S, Yu X, An YA, Zhu Y, (2018). De novo adipocyte differentiation from Pdgfrbeta(+) preadipocytes protects against pathologic visceral adipose expansion in obesity. Nat. Commun. 9, 890. 10.1038/s41467-018-03196-x. 29497032 PMC5832777

[31] Xia JY, Sun K, Hepler C, Ghaben AL, Gupta RK, An YA, Holland WL, Morley TS, Adams AC, Gordillo R, (2018). Acute loss of adipose tissue-derived adiponectin triggers immediate metabolic deterioration in mice. Diabetologia 61, 932–941. 10.1007/s00125-017-4516-8. 29224189 PMC5844860

[32] Zhao S, Mugabo Y, Iglesias J, Xie L, Delghingaro-Augusto V, Lussier R, Peyot ML, Joly E, Taïb B, Davis MA, (2014). alpha/betaHydrolase domain-6-accessible monoacylglycerol controls glucose-stimulated insulin secretion. Cell Metab. 19, 993–1007. 10.1016/j.cmet.2014.04.003. 24814481

[33] Wang S, Zhu Q, Liang G, Franks T, Boucher M, Bence KK, Lu M, Castorena CM, Zhao S, Elmquist JK, (2021). Cannabinoid receptor 1 signaling in hepatocytes and stellate cells does not contribute to NAFLD. J. Clin. Invest. 131, e152242. 10.1172/JCI152242. 34499619 PMC8592555

[34] Zhu Y, Zhao S, Deng Y, Gordillo R, Ghaben AL, Shao M, Zhang F, Xu P, Li Y, Cao H, (2017). Hepatic GALE Regulates Whole-Body Glucose Homeostasis by Modulating Tff3 Expression. Diabetes 66, 2789–2799. 10.2337/db17-0323. 28877911 PMC5652600

