# Supplementary material for: The adiponectin-PPARγ axis in hepatic stellate cells regulates liver fibrosis
Source: Cell Rep. Author manuscript; Available in PMC 2025 Feb 20. (PMC11839304; doi:10.1016/j.celrep.2024.115165)
Supplement: 1 [file NIHMS2052759-supplement-1.pdf]

Cell Reports, Volume *44*

Supplemental information

The adiponectin-PPARγ axis in hepatic stellate cells regulates liver fibrosis

Shangang Zhao, Qingzhang Zhu, Wang-Hsin Lee, Jan-Bernd Funcke, Zhuzhen Zhang, May-Yun Wang, Qian Lin, Bianca Field, Xue-Nan Sun, Guannan Li, Mbolle Ekane, Toshiharu Onodera, Na Li, Yi Zhu, Christine M. Kusminski, Terry D. Hinds Jr., and Philipp E. Scherer

Supplemental Figures

Supplemental Figure 1: Characterization of Lrat-rtTA mice.

(A-C) mRNA expression of **(A)** rtTA, **(B)** Lrat, and **(C)** Adiponectin in HSCs, liver, adipose tissue, heart, kidney, cortex, hippocampus, cerebellum, duodenum, jejunum, ileum, colon, eye and testis of Lrat-rtTA mice (n = 3-5). **(D-E)** Control (Lrat-rtTA) and LGFPTG (Lrat-rtTA+TRE-GFP) mice were fed chow+DOX for 2 weeks. **(D)** Experimental setup. **(E)** Liver immunohistochemistry for GFP and adiponectin (n =5). Scale bar represents 100 µm.. Data are displayed as mean ± SEM and analyzed by Student's *t* test or one-way ANOVA: *$p < 0.05$, **$p < 0.01$, ***$p < 0.001$.

Supplemental Figure 2: HSC depletion protects from MCD diet-induced liver fibrosis.

Control (Lrat-rtTA) and LATTAC (Lrat-rtTA+TRE-ATTAC) mice were fed chow+DOX for 2 weeks and then switched to MCD diet for another 6 weeks. **(A)** Experimental setup. **(B)** Body weight development on MCD diet (n = 6). **(C)** OGTT after 6 weeks of MCD diet (n = 6). **(D)** Liver trichrome staining after 8 weeks of MCD diet (n = 6). Scale bar represents 100 µm. Data are displayed as mean ± SEM and analyzed by Student's *t* test or one-way ANOVA: *$p < 0.05$, **$p < 0.01$, ***$p < 0.001$.

Supplemental Figure 3: Serum parameters of LAPNTG and Ctrl mice under thermoneutral housing.

(A-E) Control (Lrat-rtTA) and LAPNTG (Lrat-rtTA+TRE-Adipoq) mice were housed under thermoneutral conditions and fed HFD+DOX for 1 year to induce liver fibrosis. Circulating levels of **(A)** leptin, **(B)** cholesterol, **(C)** triglycerides, **(D)** AST, and **(E)** ALT (n = 8). Data are displayed as mean ± SEM and analyzed by Student's *t* test or one-way ANOVA: *$p < 0.05$, **$p < 0.01$, ***$p < 0.001$.

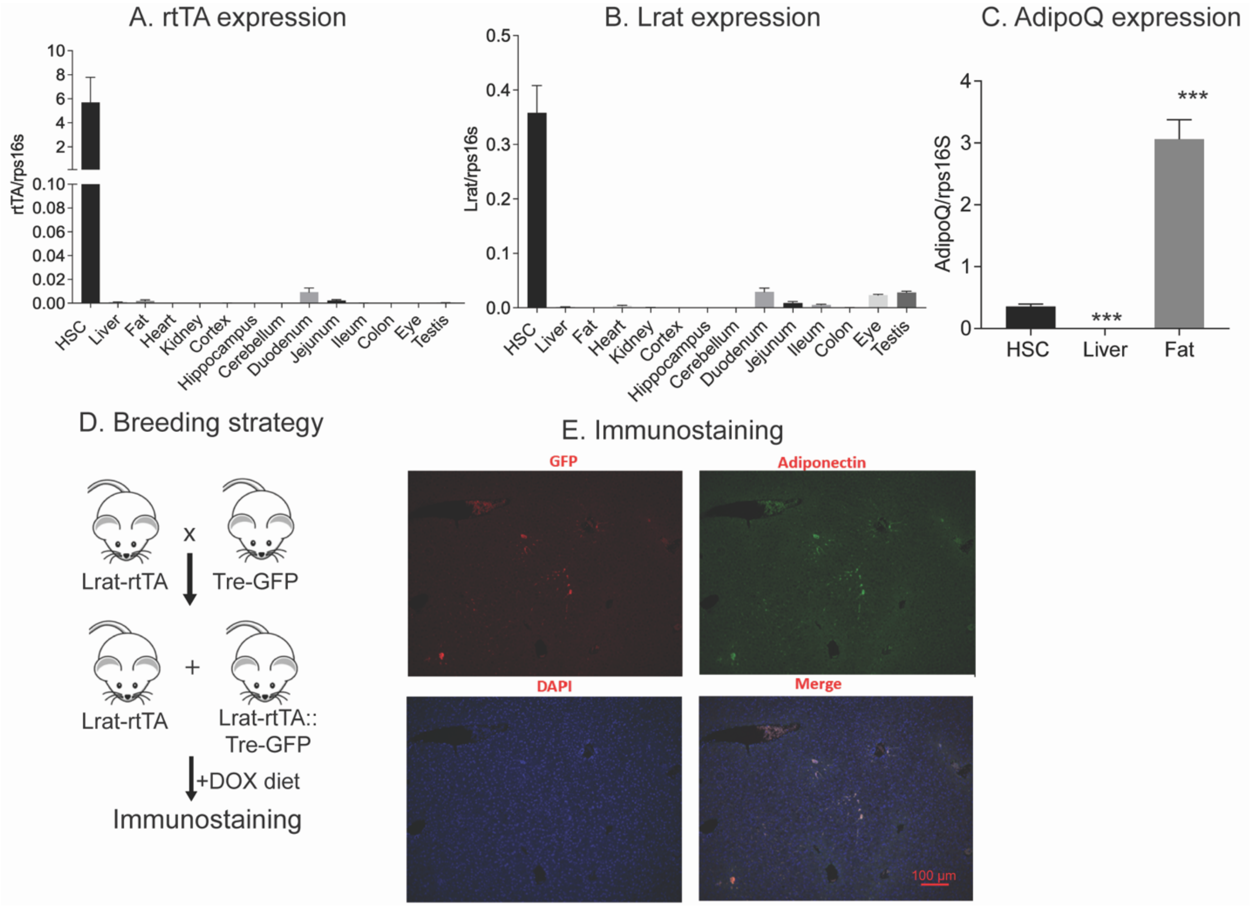

Supplemental Figure 1

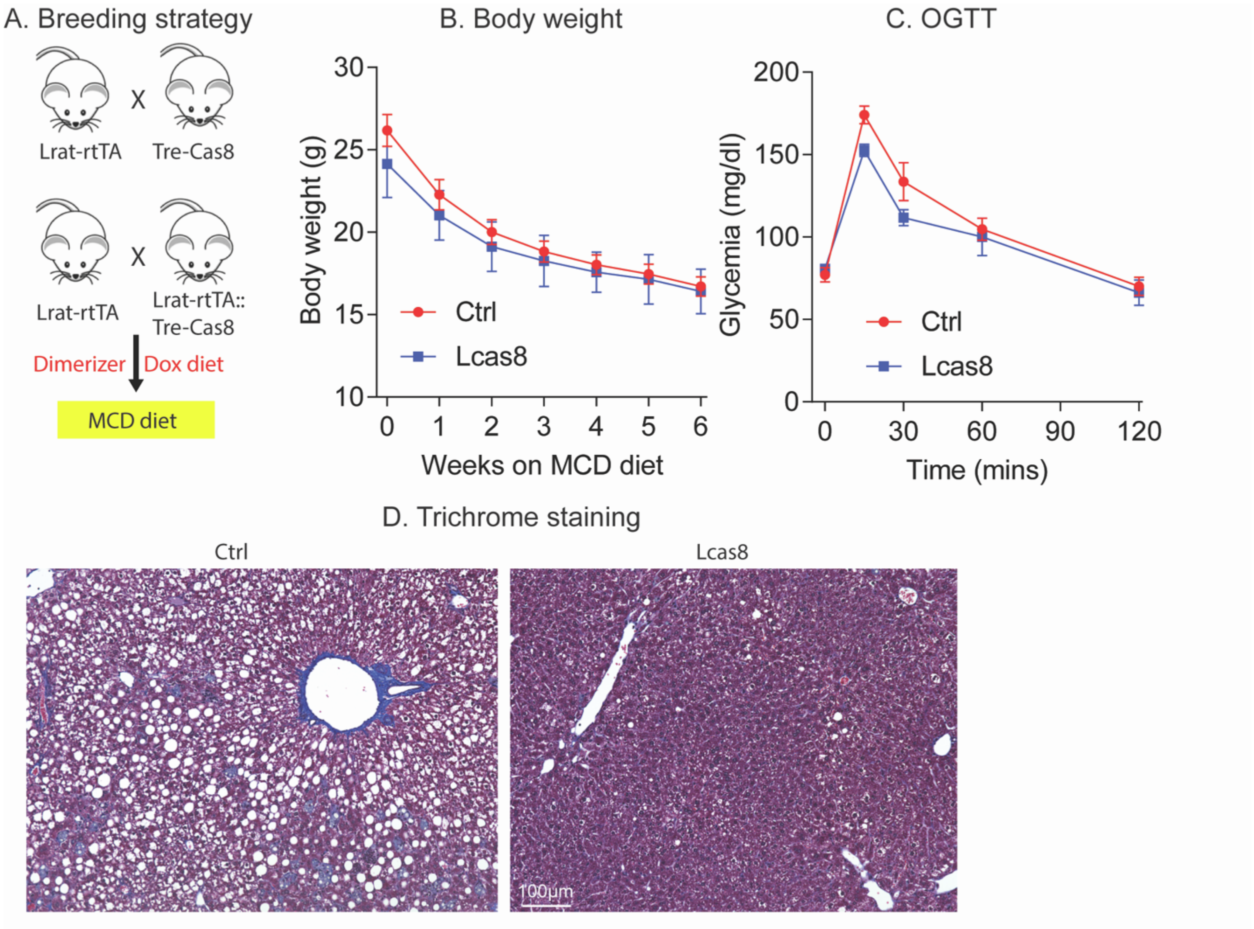

Supplemental Figure 2

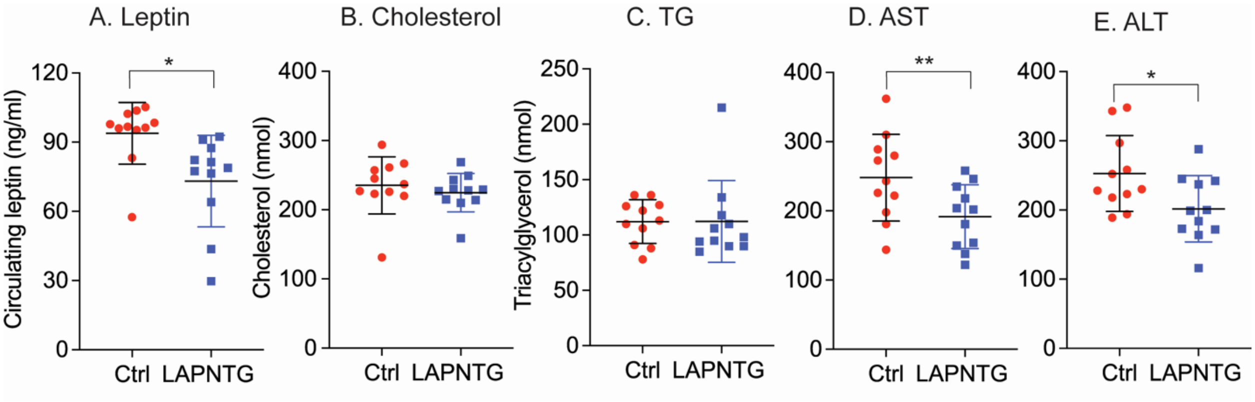

Supplemental Figure 3
